# Exploring outcome measures with cognitive stimulation therapies and how these relate to the experiences of people with dementia: A narrative literature review

**DOI:** 10.1177/14713012211067323

**Published:** 2022-01-21

**Authors:** Alison R Ward, Diana Schack Thoft, Ann Lykkegaard Sørensen

**Affiliations:** 6087 University of Northampton, Northampton, UK; 114236 University College Northern Denmark, Aalborg Oest, Denmark

**Keywords:** dementia, outcome measures, cognitive stimulation therapy, cognitive training, cognitive simulation

## Abstract

A narrative literature review was undertaken to consider the outcome measures used in research on cognitive stimulation therapy (CST), cognitive training (CT) and cognitive stimulation (CS) interventions. This review extends findings from previous reviews by including a broad range of study methodologies, both qualitative and quantitative, and explored whether participant experiences of taking part in the research are discussed. A database search identified 1261 articles matching the search criteria, with 29 included in this review. Studies tended to use the manualised CST model, with 11 other models identified. Randomised control trials were chosen as the most used method to explore impact. Across the studies, 65 different outcome measures were used with people with dementia, and only four studies used a qualitative approach. Little information is provided on the assessment process in terms of time taken, assessor, or of the experience of the person with dementia. There is heterogeneity of measures used, within and across domains, and number, and agreement or consistency of measures would provide greater comparability across CS studies. Gaps in reporting were noted on the detail of the assessment process and the experience of people with dementia taking part in this research.

## Introduction

The focus on living well with dementia has been a driver for research to understand the impact and benefit of non-pharmacological interventions, especially given that no cure for dementia is imminent (Wong & Knapp, 2020). One of the most broadly researched areas is that of cognitive stimulation (CS), which originated from reality orientation (RO) (Orgeta et al., 2015; Spector et al., 2003). This psychosocial intervention aims to support and stimulate cognitive and social functioning to improve wellbeing and quality of life (Clare & Wood, 2003; Orrell et al. 2014; Orgeta et al., 2015; Spector et al., 2010), and is usually delivered in groups. CS covers a wide range of different interventions, with the most widely known and used being cognitive stimulation therapy (CST), developed by Spector et al. (2003) and now recommended for people with mild to moderate dementia by the National Institute for Health and Care Excellence (NICE) guidance (NICE, 2018). However, other forms of CS exist, such as cognitive training (CT). Training uses task repetition to support the maintenance or training in a particular task, and is often defined as guided practice on a set of standard tasks designed to reflect particular cognitive functions such as memory or executive function (Clare & Woods, 2003, 2004).

Several systematic reviews have been conducted investigating the potential impacts of CS, in its various forms, with people with dementia. These have identified benefits in terms of quality of life, wellbeing and cognitive function (Aguirre, Woods et al., 2013a; Chao et al., 2020; Lobbia et al., 2019; Woods et al., 2006). Only one review has focused on the qualitative aspects of CST, findings from which complement the quantitative reviews (Gibbor et al., 2021). There is therefore less focus reported on the experiences of those using CST and how this develops our understanding of the impact of this intervention. In this context, this review makes three contributions. Firstly, it identifies what outcome measures CST, CS and CT use (although not to repeat previous reviews, this paper does not cover the outcome of these measures). Secondly, it evidences what CS approaches are being used and, thirdly it analyses what is reported about the impact on people with dementia taking part, that is emotionally or physically. The importance of understanding what measures are being used, and how a person with dementia may experience the assessment process, may support future studies to use similar measures, enabling comparisons to be drawn across interventions, and develop approaches that support people with dementia through the research process. This ensures that different interventions can be assessed for their potential benefits and impacts for people with dementia and that the most robust designs and approaches are being adopted (Harding et al., 2019; Harrison et al., 2016). Webster et al. (2017) argued there is a need for congruity across studies to enable comparisons and ensure the measures used are sensitive enough to identify a change. Additionally, that they are reliable and appropriate for people with dementia and do not cause undue burden or distress (Harrison et al., 2016; Webster et al., 2017).

## Methods

We undertook a narrative literature review of international peer reviewed articles to identify and critically analyse research that has been conducted on CST, CS and CT. The review was undertaken between August 2018–July 2019. The findings of the review have been informed by Ferrari’s (2015) and Baumeister and Leary’s (1997) frameworks for reporting narrative reviews. This review format was chosen as it allows for different methodological approaches to be assessed, enabling mixed methods, qualitative and/or quantitative studies to be included (Ferrari, 2015). In line with the narrative literature approach, the Mixed Methods Appraisal Tool (MMAT) was used to assess the validity and reliability of the studies (Hong et al., 2018). This tool is designed to critically appraise the quality of different research designs and methodologies. The MMAT was identified as suitable for this review which sought to include both qualitative, quantitative and mixed methods research, providing a new approach to the review of literature in this field.

**Figure 1.**
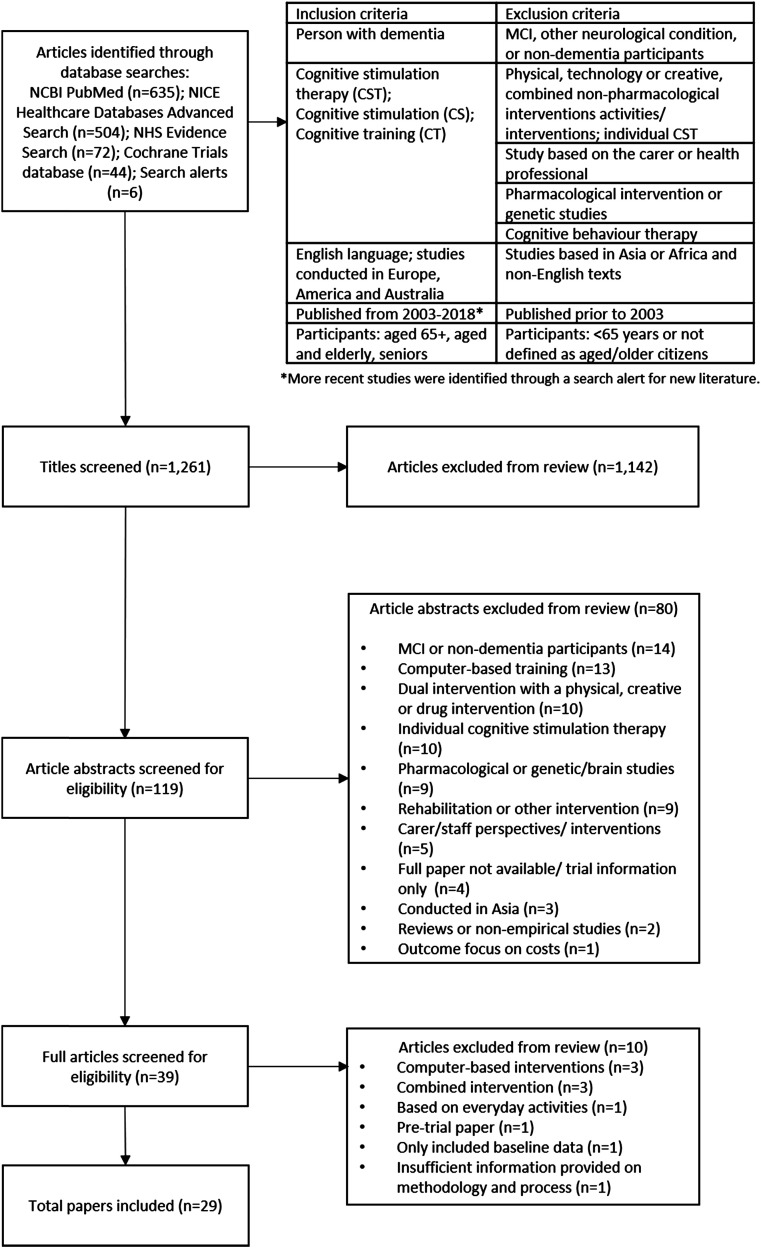
Flow chart.

To identify the literature for inclusion in this review, the following databases were used: NCBI PubMed (*n*=635); NICE Healthcare Databases Advanced Search (*n*=504); NHS Evidence Search (*n*=72); Cochrane Trials database (*n*=44). Ongoing search alerts identified nine further studies. Figure 1 includes the inclusion and exclusion criteria used to inform the initial search and identify the final articles. Studies from 2003 were included, to be in line with influential literature published by Spector et al. (2003) on their work on CST. Only fully available research texts were included, any letters, commentaries or literature reviews were excluded.

A total of 1261 articles were identified through the search. After an initial review of the titles and research origin, duplications and texts which did not meet the inclusion criteria were removed (*n*=1142). This left 119 texts to be reviewed in more detail, through an initial reading of each abstract. This enabled the research team to remove a further 80 articles, which were identified as being pharmacological or genetic studies; MCI or non-dementia participants; focused only on carer/staff perspectives; reviews or non-empirical studies; computer-based training; dual intervention with a physical, creative or drug intervention; or included individual cognitive stimulation therapy (see Figure 1). Studies from a range of contexts (including Asia) were excluded on other criteria related to cultural context and using mixed interventions. The remaining 39 articles were read and reviewed from the full text (where articles were not available to the research team, interlibrary loans were made to access full copy of all texts). Following this stage, 29 articles were identified for inclusion in this review (see Table 1 in Supplemental Material). Three articles were excluded because they included computer-based interventions, three were researching a combined intervention, one was based on everyday activities, one was a pre-trial paper and one only included baseline data. Finally, one paper was not included as it was assessed as providing insufficient information on methodology and process, as assessed by the Mixed Methods Appraisal Tool. The final 29 articles were re-read by two researchers, who completed a spreadsheet (see Table 1 in Supplemental Material) to capture the main details of each paper, under agreed headings, for example: type of study, participant demographics and outcome measures.

## Results

This review explored the measures used with people with dementia, how they experienced the assessment process and identified different cognitive training/stimulation intervention approaches

### Study type and participant demographics

Twelve of the studies used a randomised control trial methodology, and only four studies included a qualitative methodology (Bertrand et al., 2019; Kelly et al., 2017; Liu et al., 2020; Spector et al., 2011). Most studies used a control and intervention group approach, although not all were randomised and blinded for the assessors and/or research team. Of those studies using control groups (*n*=17), eight reported their control group continued to access treatment as usual (TAU) (Coen et al., 2011; Kallio et al., 2018; Mapelli et al., 2013; Middlestadt et al., 2016; Orrell et al., 2014; Spector et al., 2003; 2010; Streater et al., 2016), five engaged in other activities, such as educational activities, occupational therapy, non-cognitive activities or global stimulation (recreational activities) (Bergamaschi et al., 2013; Capotosto et al., 2017; Farina et al., 2006; Piras et al., 2017; Salotti et al., 2013). One control group accessed a different type of cognitive training as a comparison (Cavallo & Angilletta, 2017), one included a control group accessing TAU and two CST intervention groups—a standard CST group and one that included carer training (Cove et al., 2014), one included a CST group with or without a maintenance programme (Orrell et al., 2005). Finally, one control group accessed the same intervention but were a healthy cohort, without any dementia diagnosis and were compared to a group with dementia (Cavallo et al., 2016). This was included as it revealed how the intervention group with dementia responded to the intervention.

The sample size for studies varied, from an individual case study to 272 participants at baseline. The mean number of participants at baseline was 72.6. The mean age of participants was 80.6, with a mean age range from 71 to 89.3. Of the 29 studies, three did not report the gender mix. For all but five studies, there were a higher number of female participants (ranging from 55%–100%). Twelve studies included participants who predominantly had a diagnosis of Alzheimer’s disease (ranging from 52%–100%), four had a mix of dementia diagnosis, and 13 did not provide a diagnosis breakdown.

### Different approaches to cognitive training or stimulation

The most widely used intervention approach was Spector et al.’s (2003) CST model (*n*=18). Other approaches included an adapted Italian version (*n*=2) (Capotosto et al., 2017; Piras et al., 2017) and three used this in conjunction with a maintenance programme (Orrell et al., 2005, 2014; Streater et al., 2016). The timeframe for these interventions was as specified by Spector et al., with delivery of the programme over 7 weeks with 14 sessions of 45 min, and, where used, a 24 or 16-weeks maintenance programme – although two studies ran the sessions only once weekly, with one reporting extended sessions of 90 min (Cove et al., 2014; Kelly et al., 2017). Eleven other models were used, and these were based on cognitive training, cognitive rehabilitation, reality orientation and cognitive stimulation models (Table 1). Of those not using the standardised CST intervention timeframe for delivery, session times varied from weekly to three times a week for three months to up to a year (Table 1). This shows heterogeneity in how interventions are defined and/or delivered, with the greatest continuity found in the manualised CST approach developed by Spector et al. (2003).

**Table 1.** Overview of CS, CT and RO methods used.

CS/CT intervention	Summary
Cognitive training	Based on a modified version of cognitive remediation therapy (CRT) to stimulate attention, memory, cognitive flexibility and planning (Kallio et al., 2018, p.4, p.4)
Cognitive training	Session aimed to support spatial orientation, memory, attention and perception, including exercises on time and logic skills (Bergamaschi et al., 2013, p.422–3)
Reality orientation and cognitive training	Sessions included orientation to time and space, and training on memory, attention and speech (Salotti et al., 2013, p.404, p.404)
Geographical exercise for cognitive optimisation (GEO)	Cognitive training to complete a ‘puzzle like task’, for example arranging countries in the correct place on a map (cavaillo et al., 2016, p.185)
Cognitive stimulation	Sessions included orientation, stimulation exercises for memory, language, spatial and temporal orientation, attention and logic (Mapelli et al., 2013, p.266, p.266)
Cognitive stimulation	Sessions included memory and neuropsychological rehabilitation training and activities of daily living (Farina et al., 2006, p.213, p.213)
3R mental stimulation (CS)	Using reality orientation therapy, reminiscence and remotivation therapy (Mazzucchi, 2012) in each session (Spagnolo et al., 2015, p.152, p.152)
CS therapeutic sessions	Therapeutic workshops conducted with people with dementia, included guidance and informative lectures (Da cruz et al., 2915, p.451)
Cognitive rehabilitation treatment	Training on activities of daily living, executive function, memory and language. Sessions were not run with a structured schedule (Fernandez et al., 2011, p.100–101)
CS and occupational therapy (based on woods, 2004; Bottino et al., 2005)	Sessions and discussion groups to enhance cognitive and social function (Martinez et al., 2016, p.1824, p.1824)
NEUROvitals sinnreich (modified version) ()	Sessions start with a report on participants’ mood followed by exercises to support: Executive function, memory, language and cognition. Time is also provided for muscle relaxation and mindfulness and sensory stimulation (Middlestadt et al., 2016, p.256, p.256)

### Measures used to assess cognition

A total of 65 different measures were recorded, presenting great diversity. The most commonly used were the Mini-Mental State Examination (*n*=22) (MMSE – Folstein, Folstein, & McHugh, 1975), Quality of Life Alzheimer’s Disease (*n*=15) (QoL-AD – Logsdon et al., 1999; 2002), and Alzheimer’s Disease Assessment Scale – Cognition (*n*=11) (ADAS-Cog – Rosen, Mohs, & Davis, 1984).

The fewest number of validated measures used with people with dementia in any one study was two, with the most number of assessments used being 13 (Cavallo & Angilletta, 2017). The mean number was 5.5 measures (excluding proxy, caregiver or staff measures). This did not include additional measures used to assess research inclusion, or information collected on medical history, use of dementia medication or demographics.

Other research approaches were used, such as interviews with caregivers, focus groups or participant/occupational therapy observations, qualitative notes made by the CST leads (Coen et al., 2011), adherence to treatment and participant records to monitor intervention adherence (Aguirre, Hoare et al., 2013b). However, these methods were in the minority and were mostly subsidiary to the reporting of the outcome measures.

The MMSE was the most widely used measure for assessing cognition (*n*=22). Of those studies not using the MMSE as their assessment criteria, three used it as part of their inclusion criteria. Three studies (Kelly et al., 2017; Liu et al., 2020; Stewart et al., 2017) used an alternative cognitive assessment: Saint Louis University Mental Status Exam (SLUMS – Tariq et al., 2006); Montreal Cognitive Assessment (MoCa – brooke’s Cognitive Assessment III (ACE-III – Hsieh et al., 2013). Seventeen studies used the MMSE and another cognitive assessment (both global tests and individual tests). This varied, 11 used the ADAS-Cog, and seven used other cognitive assessments: Clinical Dementia Rating Scale (Hughes et al., 1982; Morris, 1993); Esame Neuropsicolgico Breve2 (Mondini et al., 2011); Milan Overall Dementia Assessment (Brazzelli et al., 1994); Mattis Dementia Rating Scale (Mattis, 1988); Short Intelligence Test; Repeatable Battery for the Assessment of Neuropsychological Status Stimulus Booklet A (Randolph et al., 1998). One study used the MoCa in conjunction with the MMSE (Kelly et al., 2017), dependent on the assessment participant’s doctors used, and MMSE scores were converted into MoCa scores for use as the primary cognitive measure.

Cognitive skills and memory were tested through a range of additional measures, which focused on different functional aspects of memory (e.g. semantic), recall, verbal fluency, orientation, visual skills, numeracy, auditory comprehension and motor skills (see Table 3 in Supplementary Material). Studies using these measures argued for the importance of measuring more specific areas of cognition and memory, as generic measures might not show any effect (Capotosto et al., 2017; Cavallo & Angilletta, 2017; da Cruz et al., 2015; Farina et al., 2006; Hall et al., 2013; Martinez-Moreno et al., 2016; Piras et al., 2017; Spector et al., 2003).

### Measuring quality of life and other aspects of living with dementia

Other domains measured included quality of life, with 15 studies using the QoL-AD, four of which used the proxy version in addition to the self-rated version (Aguirre et al., 2013b; Kelly et al., 2017; Orrell et al., 2014; Middlestadt et al., 2016). Other quality of life measures used included the Dementia Quality of Life scale (DEMQOL – Smith et al., 2005), of the two studies that used this measure, both used the proxy version (Aguirre et al., 2013b; Orrell et al., 2014), and the 15-dimensional instrument (Sintonen, 2001) proxy version (Kallio et al., 2018).

Other domains assessed included activities of daily living, anxiety, depression, communication, behaviour, loneliness, health, functional skills and quality of relationships, satisfaction, confidence and wellbeing – these were assessed using 21 different measures. In addition to those used with the person with dementia, studies also included measures of carer burden and depression (Farina et al., 2006), wellbeing and satisfaction measures with caregivers (Kelly et al., 2017), quality of caregiver’s relationships (Cove et al., 2014), and facilitator’s job satisfaction (Kelly et al., 2017).

Four studies included qualitative approaches (Bertrand et al., 2019; Kelly et al., 2017; Liu et al., 2020; Spector et al., 2011), using focus groups with caregivers and/or professionals/facilitators and interviews with people with dementia. Two raised the challenges of interviewing people with dementia, reporting they were only able to elicit generalities about attending CST groups, rather than factual information about the intervention (Liu et al., 2020; Spector et al., 2011). However, this method was discussed as providing a deeper understanding of the potential benefits of CST and how it can impact on people within the group and outside (Liu et al., 2020; Spector et al., 2011). Furthermore the inclusion of the qualitative methods supported the quantitative data findings (Kelly et al., 2017; Spector et al., 2011).

### Administration of the measures

Four studies reported on the length of time for each assessment, these were identified as 45–90 min in duration (Bergamaschi et al., 2013; Hall et al., 2013; Martinez-Moreno et al., 2016; Woods et al., 2006). Four studies reported their assessment process used individual or face-to-face interviews, with the order of the tests remaining the same across all participants (Bergamaschi et al., 2013; Hall et al., 2013; Martinez-Moreno et al., 2016; Middlestadt et al., 2016). Two studies stated that the assessments were conducted as per the instructions for each test (Hall et al., 2013; Martinez-Moreno et al., 2016) and one study reported using paper and pencil with manually recorded responses (Bergamaschi et al., 2013). A greater number of studies provided information on who had conducted the assessments, with 14 reporting these had been completed by neuropsychologists (*n*=4), occupational therapists (*n*=2), psychologists or psychology graduate (*n*=2), gerontologist (*n*=1), general practitioner (*n*=1) and researchers, PhD student or study nurses (*n*=7) – four studies used a combination of staff to undertake the assessments (Coen et al., 2011; Kallio et al., 2018; Martinez-Moreno et al., 2016; Streater et al., 2016). One study stated the assessor had been trained in undertaking tests with people with dementia (Middlestadt et al., 2016) and four specified that staff were experienced or had specialist dementia experience (Cavallo & Angilletta, 2017; Cavallo et al., 2016; Kallio et al., 2018; Martinez-Moreno et al., 2016). The qualitative studies reported details of the length of time for the focus groups and interviews and confirmed these had been undertaken by a clinical psychologist or the research team.

### Consideration of the use of measures with people with dementia

Several studies reported on the domains being measured, suggesting other factors may be more relevant or important than the current focus on cognition, such as quality of life, social interactions, individual need, self-esteem, mood, communication, care costs and doctor/hospital visits (Cove et al., 2014; Orrell et al., 2014; Middlestadt et al., 2016). These, it was suggested, may provide a more holistic understanding of the experiences and impact of CS interventions for people with dementia. The addition of qualitative notes from CST leads, which reported on the communication between group members, their enjoyment of the sessions and how this carried into other sessions, were also identified as adding valuable insight into the impact of the interventions (Coen et al., 2011), suggesting that other methods could be used to research CS.

The ‘sensitivity’ of the measures was a key area of discussion, particularly with regards to explanations for study findings. Of particular interest was the relevance of measures at different stages of dementia, for example the Functional Living Skills Assessment (Farina et al., 2010) was reported as being sensitive for measuring the everyday function of people with mild to moderate dementia (Farina et al., 2006), while the MMSE was thought to be more relevant to those in the later stages of dementia (Capotosto et al., 2017; Cove et al., 2014). Further challenges with measures were reported in small score ranges (Fernandez et al., 2011), large floor effects and lack of flexibility for participants with language difficulties (Hall et al., 2013), and administration variances and ‘subjective interpretation’ which could result in lower participant scores (Fernandez et al., 2011; Spector et al., 2010). Criticism was also reported for the ADAS-Cog and MMSE, which were considered to only assess generic cognitive change, with suggestions that specific cognitive assessments may provide more relevance (Hall et al., 2013; Spector et al., 2010). Furthermore, there may be a need to use dementia condition specific measures, as many assessments are aimed at those with Alzheimer’s disease. This was particularly relevant for quality of life and activities of daily living, as dementia specific measures may be better able to assess changes (Piras et al., 2017).

Comment on the sensitivity of measures was not limited to those assessing cognition, with the quality of life measures also being discussed, particularly in relation to the use of proxy and user versions. Whether these assessed the same domains and were understood in the same way by different participants was questioned, as differences were reported by the person with dementia and by a carer or staff member (Aguirre, Hoare et al., 2013b; Kelly et al., 2017; Woods et al., 2006). It was identified that personal issues such as stress or hope could impact on the final score (Aguirre, Hoare et al., 2013b). Questions were also raised about the congruence between measures to assess the same qualities, with particular discussion of the QoL-AD and DEMQOL, and therefore whether further research is needed to understand these measures (Aguirre, Hoare et al., 2013b). Finally, it was recommended that such measures should assess changes that result from the intervention as well as from the decline in dementia, and therefore may need to be more sensitive (Aguirre, Hoare et al., 2013b).

The challenges of interviewing people with dementia were also identified. Few suggestions were made on how to overcome these; however, one study reported that interviews had been conducted at home, around familiar objects, to support recall. Furthermore, this study specified that interviews were conducted with individuals to ensure the person with dementia was able to share their views (Liu et al., 2020).

### Impact on people with dementia

Studies commented on the appropriateness of the measures for their reliability and validity with the cohort, but not on how they might be experienced by the participants with dementia. One study described taking actions to minimise any impact on participants by limiting the time of the assessments to reduce unnecessary burden (Hall et al., 2013). No studies reported how participants had reacted during the assessment or if there had been any feedback from the participants on how it felt to be part of the study. Two studies commented that some participants had not been able to complete all of the assessments due to refusal, fatigue or difficulty retaining task instructions (Hall et al., 2013; Kallio et al., 2018). One study discussed the dropout rate from baseline to final assessment stating this was not affected by the assessments used (Spector et al., 2010).

## Discussion

There is great heterogeneity of measures and study designs used in assessing the impact of CST, CS and CT, and little agreement about which measures to use (Reilly et al., 2020). As more general reviews of psychosocial interventions have found (Harding et al., 2019; Harrison et al., 2016; Webster et al., 2017), the range of measures varies not only within a domain, such as cognition, but also in determining what domains are important to measure, with quality of life and cognition being the most commonly assessed. Webster et al.’s (2017) review of disease-modification interventions with people with dementia reported that out of 125 trials, 81 different outcome measures were used, while this current review identified 65 different measures. There is no discussion about how many measures is suitable to include in a battery of tests, this review found the number ranged from 2–13. In their recent review of outcome measures used in non-pharmacological interventions with people with dementia, Couch et al. (2020) identified that up to 21 different measures could be used. No comment is made in the studies as to the experience of people with dementia on participating in a large battery of measures, further understanding of the impact of taking part in such research may provide guidance on what number of measures is appropriate for participants, and how to ensure they are not overburdened or fatigued through participation.

The main method adopted by studies in this review was through an RCT or other quantitative control/intervention group method. Only a few of the papers used qualitative data collection as their main method. Interestingly, several papers discussed the value of including an element of qualitative feedback, either through observations or facilitator notes. This suggests there is the potential to develop a mixed methods approach, with complimentary qualitative and quantitative data to provide a more holistic understanding of how CS interventions may have an impact and are experienced by people with dementia. Also, of note is that the more recent studies included in the review were using a mixed methods approach.

However, this contradicts some author’s views (Orrell et al., 2005; Spector et al., 2010; Tárraga et al., 2006) who suggested that studies should be aligned to the methods and timescales of clinical drug trials. By using the same measures as clinical trials, it becomes easier to compare across studies and it may perhaps lead to CS interventions being more efficacious when the same methodological approaches are used (Tárraga et al., 2006). However, there was a lack of consensus as several authors suggested the relevance of measuring other domains when evaluating CS, and that not all relevant domains are included by the most frequently used outcome measures (Cove et al., 2014; Gibbor et al., 2021; Middlestadt et al., 2016; Viola et al., 2011). Qualitative approaches may ensure the nuances of people with dementia’s experience are included, and support understanding of relevant unmeasured domains, such as wider social benefits, confidence, self-esteem or engagement in sessions, together with measuring reduction in health and social care costs, for example general practitioner visits and hospital admissions (Cove et al., 2014; Spector et al., 2011; Viola et al., 2011).

Reviews on measures used in dementia research (Harrison et al., 2016; Webster et al., 2017) comment on the sensitivity of the different tests, particularly in relation to detecting small changes in those with milder symptoms of dementia, an aspect also identified in this current review. Harrison et al. (2016) notes that some commonly used measures are not designed to be used in RCTs, with particular mention to the MMSE – one of the most widely used standardised measures (Couch et al., 2020). The strength of the MMSE is in its wide use, across many different cohorts and conditions, and therefore allows for comparisons across studies (Carnero-Pardo, 2014). However, it has been criticised for not being sensitive enough to use in dementia studies or for different types of dementia (Carnero-Pardo, 2014). Harrison et al.’s (2016) findings support the criticisms of sensitivity for the MMSE, but also other cognitive measures, including the ADAS-Cog, both measures recommended through Webster et al.’s (2017) review.

It is challenging for studies to decide on the best measures to use, particularly when so many measures of cognition exist and there is little agreement on which to use. Couch et al. (2020) identified over 40 in their review of outcome measures. Researchers are trying to identify a standardised set of measures to use in dementia research, Core Outcome Measures in Effectiveness Trials COMET (2020) are reviewing this (http://www.comet-initiative.org/), with a recent study identifying four core areas of importance: Friendly neighbourhoods and home, independence, self-management of symptoms, and quality of life (Reilly et al., 2020). While the International Consortium for Health Outcome Measures (2020) suggest the following measures: Neuropsychiatric Inventory, Montreal Cognitive Assessment, Bristol Activity of Daily Living, Quality of Life-AD and Quality of Wellbeing Scale-Self Administered, EuroQol-5D and the Clinical Dementia Rating. A review of outcome measures by INTERDEM (Moniz-Cook et al., 2008) identified 22 measures for use in dementia research and further advise is offered by the NIH toolbox for Assessment of Neurological and Behavioural Function (Gershon et al., 2013), which provides a range of measures designed to address some of the criticisms of ceiling effects, sensitivity that many studies encounter with their measures, and to provide a uniformity to what and how change can be measured effectively. As part of a wider study speaking with people with dementia, caregivers and experts in dementia, Webster et al. (2017) concluded that biomarkers should also be used. While this current review did not include research that had used biomarkers as core outcome measures, it should be noted that none of the studies used an MRI or other biological test in conjunction with the standardised measures, although recommendations were made to use these in future research.

There is little information on the way assessments are conducted, by who and what training or review of the process is carried out – especially if more than one individual is conducting the assessments. Greater clarity of this process would aid others in repeating similar studies and identifying potential ethical dilemmas when assessing people with dementia. Harrison et al. (2016) makes recommendations for more information to be provided on how tests are used. The present review found that little information was provided about the application of the tests. This is an area that could be developed further to ensure replicability and robustness of approach. Furthermore, there was little information reported on the impact of the measures used on people with dementia, some commented on the length of time and the potential for burden but not on how they were experienced by people with dementia, or of ethical discussions on potential burden of participation. More research could be conducted in this area to understand how people with dementia experience being part of a battery of tests and what occurs in the process.

Webster et al.’s (2017) participants with dementia and their caregivers stated that time was a concern in taking part in such studies, both the time they take and the travel implications. If research is to continue to encourage people with dementia to participate, an understanding of what takes place in the assessment process could provide researchers with greater knowledge of how best to include and support participants. Thus, not overburdening them. This could be helpful in understanding how many tests may be suitable to conduct, the time these should take and how to support someone with dementia. As Webster’s participants commented, they could experience anxiety and be demoralised in taking part, an ethical consideration that all studies should minimise. The recent rise in using qualitative methods within CS studies provides an additional way of exploring the impact of CS. For example through using validated observational methods or interviews, as this may encourage and support engagement (Liu et al., 2020) and provide a sense of worth and personhood in the process (Gibbor et al., 2021). However, a qualitative approach per se does not guarantee ethical inclusion of people with dementia in research. Here it is also important to judge what are the best ways to include and support the participants.

One of the main dilemmas in researching people with dementia is in deciding on the best method. The papers in this review questioned the sensitivity of the measures used, the consistency of response from people with dementia across time periods, and whether observations may provide a more sensitive approach. There is a question mark over the reliance on validated measures, and suggestions that these may miss other important factors such as social interactions, access to healthcare, self-esteem, social health and level of independence (Cove et al., 2014; Harrison et al., 2016; Orrell et al., 2014; Middlestadt et al., 2016; Reilly et al., 2020). There may also be a concern with selection bias, that research is focussing on particular types of participants, namely older people with Alzheimer’s disease, while participants with different types of dementia or symptoms, such as aphasia, are often excluded from research (Dalemans et al., 2009). Furthermore, research should be encouraged to seek the views of people with dementia and their caregivers to ensure that the outcome measures are relevant and important to their participants (Harrison et al., 2016). Harrison et al. (2016) also considered that research should capture people’s lived experience. In their study to review core effect measures in dementia research, Webster et al. (2017) spoke with people with dementia and their caregivers to identify what measures would be important to them. They found that individual differences and personality traits were thought to have an impact on the way a person responded, and therefore finding a way to capture this would be important. Harding et al. (2019) reports that what may be important for the health and research community, and the intervention, may not be for the person with dementia and their caregivers. They are rarely included in decision making about what outcome measures to use, and this is an area that could be developed in future research, particularly given the shift in thinking to involve the person with dementia in all aspects of research (Morbey et al., 2019).

### Study limitations

While every endeavour was taken to identify all relevant papers in this review, the databases chosen may have excluded some papers, particularly qualitative papers. Cross references with other literature reviews has identified that key papers have been included, and an alert for new articles resulted in additional papers being included in the review. The decision to exclude papers that focused on cultural contexts of the use of CST may have been limiting and future reviews should consider their inclusion, especially considering the growing interest in use of CST in different cultural settings and innovative use of methodological approaches in researching these, for example through the use of MRI scans. The wide scope of the review may be considered a limitation in drawing comparisons across wide ranging study methodologies and interventions. However, to our knowledge, this is the first literature review to make such comparisons in this expanding area of research. The nature of a narrative literature review has been criticised for its, often, un-systematic approach (Naveed et al., 2016), this paper adopted a robust approach to its review of the papers and undertook a replicable approach in the search of the literature, with specified search terms, and inclusion and exclusion criteria used to limit bias in the selection of articles. The use of the MMAT also provided a way of assessing the suitability and the quality of papers included in the review. Each paper was read by two reviewers to ensure accuracy of information recorded and assessed.

## Conclusion

This review found a high rate of heterogeneity in relation to the measures (type and number) and methods used when evaluating the effect of CS. There exist differences of opinion on the best measures to use, presenting challenges for researchers to know what may be the most appropriate measure to use and domains to assess. The current focus is to measure cognition and quality of life, but other constructs (e.g. social, confidence and wellbeing) were identified as important, as well as giving consideration of the person with dementia and/or their caregiver’s views on what should be measured. Greater agreement would support future research in this field and enable valuable comparisons across studies. This discussion is made more complex with suggestions to align research with clinical drug trials in terms of methods and approach, and by conflicting by recommendations to include qualitative elements within the research, that could provide a wider understanding of the impact of CS interventions. One of the gaps this review identified was with the level of detail provided on the assessment process, with few studies reporting who conducted, for how long and what impact the assessment process took on the person with dementia. Further research on what occurs in the assessment process may support discussions on what and how many measures to use, as this could provide valuable insight into the way a person with dementia experiences and responds in the assessment and can be supported to avoid overburden, and how the process is conducted. The development of guidelines for dementia researchers could also provide information on appropriate length of time and number of measures, provide a list of recommended measures and mixed method approaches that could support future research in this area. If such guidelines were coproduced with people with dementia, family and service representatives, researchers and service commissioners it could provide a valuable resource that would meet the needs of those designing and conducting research to ensure robust research methodologies are being used in a way that does not overly burden those taking part in the research.

## Supplemental Material

sj-pdf-1-dem-10.1177_14713012211067323 – Supplemental Material for Exploring outcome measures with cognitive stimulation therapies and how these relate to the experiences of people with dementia: A narrative literature review Click here for additional data file. Supplemental Material, sj-pdf-1-dem-10.1177_14713012211067323 for Exploring outcome measures with cognitive stimulation therapies and how these relate to the experiences of people with dementia: A narrative literature review by Alison R Ward, Diana Schack Thoft and Ann Lykkegaard Sørensen in Dementia.

sj-xlsx-2-dem-10.1177_14713012211067323 – Supplemental Material for Exploring outcome measures with cognitive stimulation therapies and how these relate to the experiences of people with dementia: A narrative literature review Click here for additional data file. Supplemental Material, sj-xlsx-2-dem-10.1177_14713012211067323 for Exploring outcome measures with cognitive stimulation therapies and how these relate to the experiences of people with dementia: A narrative literature review by Alison R Ward, Diana Schack Thoft and Ann Lykkegaard Sørensen in Dementia.
