# Supplementary material for: Exploring outcome measures with cognitive stimulation therapies and how these relate to the experiences of people with dementia: A narrative literature review
Source: Dementia (London). 2022 Jan 21;21(3):1032–49. doi: 10.1177/14713012211067323 (PMC9198284; doi:10.1177/14713012211067323)
Supplement: sj-pdf-1-dem-10.1177_14713012211067323 – Supplemental Material for Exploring outcome measures with cognitive stimulation therapies and how these relate to the experiences of people with dementia: A narrative literature review [file sj-pdf-1-dem-10.1177_14713012211067323.pdf]

Table 3: Validated measures and other assessments

Method / Validated Measure	No Studies
Mini Mental State Examination (MMSE; Folstein et al., 1975)	22
Quality of Life-Alzheimer's Disease (QoL-AD; Logsdon et al., 1999) (4 proxy version)	15
Alzheimer's Disease Assessment Scale – Cognition Subscale (ADAS-Cog; Rosen et al., 1984)	11
Neuropsychiatric Inventory (NPI; Cummings et al., 1994) (1 used Nursing Home Version)	7
Cornell Scale for Depression in Dementia (CSDD; Alexopoulos et al., 1988)	6
Clinical Dementia Rating (CDR; Hughes et al., 1993)	5
Geriatric Depression Scale (GDS; Yesavage et al., 1982-1983)	5
Activities of daily living; instrumental activities of daily living (IADL; Lawton & Brody, 1969)	5
Clifton Assessment Procedures for the Elderly – Behaviour Rating Scale (CAPE-BRS; Pattie & Gilleard, 1979)	4
Digit Span (Backwards and forwards) (De Beni, Borella et al., 2008)	4
Interviews	4
Alzheimer's Disease Cooperative Study – Activities of Daily Living (ADCS-ADL; Galasko et al., 1997)	3
Focus groups	3
Holden Communication Scale (Holden & Woods, 1982)	3
Rating Anxiety in Dementia (RAID; Shankar et al., 1999)	3
Rivermead Behavioural Memory Test RBMY; de Wall, 1994; Wilson et al., 1990)	3
Token Test (Spreen & Benton, 1969; 1977)	3
Verbal fluency for Letters and Categories (Brucki & Rocha, 2004)	3
6-item social and emotional loneliness scale (De Jong & Van Tilburg, 2006; adapted by Borella)	2
Brixton test	2
Clinical Insight Rating scale (CIR; Zanetti et al., 1997)	2
Dementia Quality of Life scale (DEMQOL; Smith, Lamping, & Banerjee, 2005) (1 proxy version)	2
Disability Assessment for Dementia (DAD; Gélina et al., 1999)	2
Esame Neuropsicologico Breve 2 (ENB2; Mondini et al., 2011)	2
Graded Naming test	2
Hayling Sentence Completion test	2
Hospital Anxiety and Depression Scale (HADS; Zigmond & Snait, 1983)	2
Milan Overall Dementia Assessment (MODA; Brazzelli et al., 1984)	2
Narrative language test (language) (Carlomagno et al., 2013)	2
Observation	2
Short Intelligence Test	2
Two-syllable word repartition	2
Visual Object and Space Perception Battery	2
Addenbrooke's Cognitive Examination III (ACE-III; Hsieh et al., 2013)	1
Attentional Matrices (Spinnler & Tognoni, 1987)	1
Basic Activity of Daily Living (ADL; (Katz et al., 1963)	1
Behavioural Pathology in Alzheimer's Disease Rating Scale (Behave-AD scale; Reisberg et al., 1997)	1
Boston Naming Test-2 (BNT-2; Kaplan et al., 2001)	1
Carer measures: Burden Inventory and Beck Depression Scale (Beck et al., 1961); Caregiver Burden Inventory (CBI; Novak & Guest, 1980)	1
Clock Drawing Test (CDT) - visio spacial skills, executive function and memory (Atalaia-Silva &	1

Lourenço, 2008)	
Cumulative Illness Rating Scale (CIRS; Linn et al., 1968)	1
D-KEFS Verbal Fluency (Delis et al., 2001)	1
Facilitator checklist of the key principles of CST	1
Functional Living Skills Assessment (FLSA; Farina et al., 2010)	1
Katz scale daily living (Katz; Lino et al., 2008)	1
Lawton Scale (Santos & Virtuoso Júnior, 2008)	1
Lifestyle Activities Questionnaire (Carlson et al., in press)	1
Likert scales for person with dementia: cognitive ability, satisfaction with cognitive performance, and well-being	1
Likert scales for person with caregivers: pwd cognitive ability, wellbeing, level of engagement, confidence and satisfaction	1
Likert scales for person with facilitators: Job satisfaction and level of engagement and satisfaction	1
Mattis Dementia Rating Scale (MDRS; Mattis, 1988)	1
Memory Awareness Rating Scale-Functioning Subscale (MARS-F; Clare et al., 2002) and proxy version	1
Modified Rey's Complex Figure (Becker et al., 1987)	1
Montreal Cognitive Assessment (MoCa; Nasreddine et al., 2005)	1
Nurses' Observation Scale for Geriatric Patients (NOSGER; Spiegel et al., 1991)	1
Qualitative notes from the CST leads	1
Quality of Caregiver and Patient Relationship (QCPR; Spruytte et al., 2002)	1
Repeatable Battery for the Assessment of Neuropsychological Status Stimulus Booklet A (RBANS; Randolph et al., 1998)	1
Review Barcelona Test (TB-R; Peña-Casanova, 2005)	1
Revised Memory and Behaviour Problems Checklist (RMBPC; Teri et al., 1992)	1
Rey figure copy and recall (Bertolani et al., 1993)	1
Saint Louis University Mental Status Exam (SLUMS; Tariq et al., 2006) cognitive assessment	1
Timed tests encompassed letter (F, A, S) and category (animals) (Novelli et al., 1986)	1
Trail Making (Reitan and Wolfson, 1992)	1
Verbal Fluency Test (VFT; Brucki & Rocha, 2004)	
Verbal Fluency (animals) (Newcombe, 1969)	
Vocabulary subtest of the Shipley Institute of Living Scale (Shipley, 1967)	1
Volitional Questionnaire (Chern, et al., 1996)	1
Wechsler Adult Intelligence Scale 3rd Edition (WAIS-III; Wechsler, 2001)	1
Wechsler Test of Adult Reading (WTAR; Wechsler, 2001)	1
WMS-III Digit Span (Wechsler, 1997)	1
WMS-III Logical Memory (Wechsler, 1997)	1
WMS-III Information and Orientation (Wechsler, 1997)	
WMS-III Visual Reproduction (Wechsler, 1997)	1
15-dimensional instrument (15D), to assess HRQoL proxy version (Rosen et al., 1984; Sintonen, 2001)	1
